# Cultural Framing and the Impact On Acute Pain and Pain Services

**DOI:** 10.1007/s11916-023-01125-2

**Published:** 2023-07-05

**Authors:** Rahel Rogger, Corina Bello, Carolina S. Romero, Richard D. Urman, Markus M. Luedi, Mark G. Filipovic

**Affiliations:** 1 Department of Anaesthesiology and Pain Medicine, Inselspital, Bern University Hospital, University of Bern, Bern, Switzerland; 2 Anesthesia, Critical Care and Pain Department, Hospital General Universitario de Valencia, Universitad Europea de Valencia, Valencia, Spain; 3 Department of Anaesthesiology, The Ohio State University, Columbus, OH USA; 4 Department of Anaesthesiology and Pain Medicine, Cantonal Hospital of St. Gallen, St. Gallen, Switzerland; 5 Department of Anaesthesiology and Pain Medicine, Pain Center, Inselspital Bern, Freiburgstrasse 18, 3010 Bern, Switzerland

**Keywords:** Culture, Acute pain, Pain services, Pain management, Cultural competence

## Abstract

**Purpose of review:**

Optimal treatment requires a thorough understanding of all factors contributing to pain in the individual patient. In this review, we investigate the influence of cultural frameworks on pain experience and management.

**Recent Findings:**

The loosely defined concept of culture in pain management integrates a predisposing set of diverse biological, psychological and social characteristics shared within a group. Cultural and ethnic background strongly influence the perception, manifestation, and management of pain. In addition, cultural, racial and ethnic differences continue to play a major role in the disparate treatment of acute pain.

**Summary:**

A holistic and culturally sensitive approach is likely to improve pain management outcomes, will better cover the needs of diverse patient populations and help reduce stigma and health disparities. Mainstays include awareness, self-awareness, appropriate communication, and training.

## Introduction

Pain is “an unpleasant sensory and emotional experience associated with, or resembling that associated with, actual or potential tissue damage” [1]. It is a universal human experience that accompanies people throughout their life [2].

The management of acute and chronic pain in both the perioperative and longitudinal settings are two central pillars of anesthesiology [3, 4]. Pain accounts for very high healthcare costs worldwide each year. According to the United States National Academy of Sciences, the estimated cost in 2010 was $560 billion to $635 billion per year, due to direct health care costs and lost productivity [5]. America is no exception, as similar data are available from European countries [6].

Simply put, pain perception from a mechanistic point of view is the activation of nociceptors of nerve fibers by a stimulus and the transmission up to the central nervous system (CNS) through peripheral nerves and the spinal cord [7]. Within the CNS, the stimulus is directed to different areas of the brain to process the incoming information [7]. Analgesics that interact with nociceptive pathways are among the most commonly prescribed drugs, with a prevalence of 20–60% in European countries and rising [8]. However, pain has been recognized as a much more complex concept in recent decades and the simple administration of analgesics does not meet the expectations of modern pain management, especially in chronic pain [9]. In light of the opioid endemic, one of the most alarming public health issues [10], a paradigm that has been strongly advocated for in chronic pain [9], has now gained attention in acute pain also: Optimal pain management requires a thorough understanding of all factors contributing to pain in the individual patient and a personalized approach to pain management is critical.

The variability and heterogeneity of individual pain responses has been recognized for nearly 80 years [11]. Additional research has shown that attention as well as emotional and cognitive components influence the quality and quantity of perception of a painful stimulus [7, 12]*.* Most interestingly, even a complex relationship between the psychosocial environment and the perception and experience of pain can be observed, as pain can be “socially modulated” [13]. These are just two examples of a wide range of biological, psychological and sociological factors that influence individual pain perception and processing.

It is impossible for the responsible clinician to assess and evaluate all of those parameters for each individual patient, especially in anesthesiology, where the doctor-patient interaction is typically brief. However, the individual factors are often highly interrelated, and patterns can be identified within a group or a society [14]. This is often referred to as “culture”. As Narayan put it, because humans are cultural beings, the groups to which we belong (ethnic, religious, geographic, socioeconomic and others) influence the way we think, what we expect and what is “right” [15]. As a result, an individual’s cultural and ethnic background strongly influences the perception, manifestation, and management of pain [2]. Considering global migration, the likeliness to treat a diverse and culturally divergent patient population is ever increasing [16]. Understanding the meaning of pain experienced within the cultural construct is essential to the physician–patient relationship and for optimal treatment.

Therefore, the purpose of this review is to examine the influence of culture and related factors on pain. First, we will attempt to further define culture and its importance in pain research, then describe individual factors within culture and finally derive possible consequences for pain management.

## The Definition of Culture in the Context of Pain

The study of pain is challenging to the researcher due to its individuality and subjectivity [14]. In addition, a myriad of complex and dynamic factors influences the perception and processing of pain. In an attempt to integrate multiple components, the biopsychosocial model postulates that biological, psychological and social factors all play a significant role in health and disease – which can be very well applied to pain [14]. Culture, in turn, is defined as “the set of distinctive spiritual, material, intellectual and emotional features of society or a social group, that encompasses, not only art and literature, but lifestyles, ways of living together, value systems, traditions and beliefs” [17]. In the context of pain, culture integrates both modifying markers and mechanisms, which are again not isolated but constantly interact and interrelate, due to the vague and heterogeneous definition [14]. Thus, culture simply attempts to subsume societies or social groups with similar predisposing characteristics [17].

This categorization, which may simplify the practitioner’s life, carries risks. First, while culture may include important predisposing factors, a cultural construct can never capture the complexity and true beliefs of an individual patient. Second, culture includes racial and ethnic background, religious beliefs, and socioeconomic affiliation. Talking about race and ethnicity and combining it with categorization can lead to discrimination and racism [18]. For example, a recent study showed that White medical students and laypersons endorsed the belief that biological differences (thicker skin) are responsible for a higher pain tolerance in Black individuals [19] – evidence that false beliefs and misconceptions continue to exist.

To avoid discrimination and racism, we adhere to a recent statement issued by Palermo et al. [18] and use language and terms that are inclusive, non-judgmental and bias-free based on the APA Style and Grammar Guidelines [20]. Further categorization and elucidation of group differences is intended to promote culturally competent clinical care and ultimately to address and reduce disparities in pain management among ethnically/racially diverse groups [21]. In the following sections, we describe the influence of various biopsychosocial factors related to culture on pain perception, processing and management.

## Influence of Culture on Pain Perception and Processing

From a biological perspective, genetic contributions to the experience of pain have been identified and found to vary by gender and ethnicity. Accordingly, the altered pathways may account for differences in pain response. Among the genes associated with pain, the one that encodes catechol-O-methyl-transferase (COMT) is particularly well known [14]. COMT is responsible for the metabolism of catecholamines [14]. So far, three COMT haplotypes were discovered, which are associated with global pain sensitivity [14]. These findings suggest that COMT is an important factor in the modulation of pain responses [22••]. Another gene of interest is that encoding the mu-opioid receptor (OPRM1) [23••]. The A118G single nucleotide polymorphism (SNP) of OPRM1 has been associated with group differences in response to experimental pain [22••]. This variation could also be observed among different racial/ethnic groups. For example, Whites with the minor allele exhibited reduced sensitivity to multiple experimental pain measures, while an opposite direction of the effect was observed in Hispanics [14]. Similar bidirectional findings have been reported for both COMT and OPRM1 in relation to gender [14]. According to some studies, chronic pain seems to be more prevalent among women than men [24]. In general, women appear to be more sensitive for standards measurements of experimental pain [14]. Many explanations for these findings have been discussed, including the biological differences but also cognitive influences and social factors such as stereotypical gender roles [24].

A few studies have applied standardized pain stimuli to groups of individuals from different ethnic backgrounds [25-27]. In these studies, Hispanics had a lower pain threshold than Whites suggesting that Hispanics may be sensitive to pain than Whites [25, 27]. Interestingly, the threshold for heat as a painful stimulus did not differ between groups, while the intensity rating on a pain scale for the same stimulus showed differences—suggesting that the perception or rating of pain differed between the groups [25]. In another study, comparing international participants (Italians, Swedes and Saudis), Italian women had the highest pain score of all these groups – again revealing biological or cultural differences in either the perception or reporting of pain [26].

Several recent studies have examined whether race and ethnicity influence pain perception. For example, a number of studies have shown overall higher pain reports in Black patients compared to White patients [28-32]. Importantly, some of the observed differences were no longer significant, after adjustment for demographic and psychological health variables as well as socioeconomic and physical health factors [30]. Asian and Hispanic women also had higher pain scores compared to White women [32]. According to a recent meta-analysis African-Americans, Asians and Hispanics may have a higher pain sensitivity compared with Non-Hispanic-Whites [33].

The explanations and possible mechanisms underlying racial/ethnic group differences pain are most certainly multifactorial. For example, brain imaging has shown structural differences in pain-related brain areas between White and Black participants, suggesting some mechanistic variation [33••]. But even these differences might be related to socioeconomic status and lack of access to adequate health care, as is likely the case for much of the reported differences.

There is substantial evidence that lower socioeconomic status is associated with poorer health outcomes [31, 35]. Consistent with the findings above, Blacks have been found to experience higher rates of discrimination and stressful or traumatic life events than Whites. In turn, higher levels of discrimination are associated with a higher prevalence of pain and worse pain management [22, 36••]. Psychological factors and stress in general have a significant impact on the chronicity of pain [14, 34••]. Individuals with chronic pain conditions typically report higher levels of psychological distress, experience greater life stress and suffer from a greater number of non-pain somatic symptoms compared to those without chronic pain [14]. Again, pain experience, structural changes in pain-related brain regions and resilience have been linked to ethnic/racial and sociodemographic differences [34••, 37, 38, 36••, ]. In addition, a combination of population-specific genetic variants and environmental factors influencing gene expression might explain the variability in drug response between patients of different racial and ethnic backgrounds [39].

In summary, genetics and other biological factors capable of modifying an individual’s experience of pain are currently under investigation. However, most cultural differences appear to be due to sociodemographic factors and life stressors, which in turn may be responsible for both psychological and bio-structural changes in pain perception. Cultural factors may also influence expression and reporting, rather than the sensory perception of pain.

## Influence of Culture on Pain Expression, Communication and Coping

### Expression of Pain

Cultural subgroups differ in how they express their pain. While some cultural groups may avoid vocalizing pain through moaning, crying or by facial expressions, others exhibit more expressive behaviors in response to painful stimuli [15]. There are also differences in seeking care or attention, with some preferring to be left alone and cope with pain without asking for care [15]. On the other hand, members of other cultures have learned to manage and relieve pain by screaming and by seeking attention and support [15]. One setting, in which intracultural differences in pain expression have been studied extensively is labor pain. For example, some Muslim women might express their pain more actively by screaming and crying, than women who self-identify as Christians [40]. Religious beliefs in general can have an impact on an individual's perceptions, emotions, and behaviors and can significantly affect health, pain sensitivity and treatment outcomes [41]. Najem et al. [41•] even found an association between religiosity and several domains of pain such as pain intensity, disability, and pain-related cognitions or emotions in people with chronic musculoskeletal pain. However, the evidence for the association was rather weak. A language barrier was also associated with greater pain expression in two studies, demonstrating the interaction of expression and communication [40, 42].

### Reporting and Communicating Pain

Not surprisingly, the lack of a common language between healthcare providers and their patients can lead to difficulties in exploring the individual level of pain. Significant difficulties arise from assessing patients in a non-native language, coupled with the level of proficiency [43]. Even with the assistance of an interpreter, communication barriers may persist [44]. This may be explained by the fact that communication differences occur not only when there is an obvious language barrier but also between people who speak the same language, due to differences in vocabulary or alternative interpretations [15]. For example, one study showed that the greater the cultural distance between a woman and her midwife, the greater the likelihood of misinterpretation of the women’s experience of labor pain [44]. Another study by Lor et al. [46] showed that both language and race affect the likelihood of reporting any pain and pain intensity: Consistent with other studies, Asian race was associated with decreased odds of reporting any pain and contributed to lower pain severity, which may be due to cultural differences [47]. Namely, a study of Chinese cancer patients concluded, that a patient from the Chinese culture who is experiencing pain, is more likely to endure the pain and not report it to a health care provider until the pain becomes unbearable [48]. Patient concerns have also been shown to significantly influence self-reported pain, with over-reporting to increase provider belief/responsiveness and out of fear of future pain, which may function in a similar way for overexpression [49].

### Concepts of and Coping with Pain

Different cultural groups are known to have different perceptions and beliefs about illness and pain. For example, there is some evidence that individuals from Brazil experiencing chronic fatigue are more likely than their British counterparts to attribute their fatigue to physical causes. Likewise, Spanish women with fibromyalgia report higher levels of negative views about the condition, whereas Dutch women with fibromyalgia exhibit more positive beliefs about its controllability [50]. The exploration of different concepts is clinically highly relevant, as it leads to greater acceptance of certain pain management interventions compared to others [14, 50-52]. It will also influence the way patients manage their pain. For example, there is moderate evidence that Blacks are more likely to use more praying, hoping, and emotion-focused coping strategies in comparison to Whites [50, 51]. Whites, on the other hand are more likely to more frequently ignore pain and to prefer relaxation techniques [50].

In summary, culture influences all dimensions of communication about pain. Culture shapes how individuals experience and respond to pain including their propensity to seek treatment and when to do so [15]. This is important, because misinterpretation of pain leads to inferior treatment [32].

## Interaction of Patients and Health Care Providers with Different Cultural Backgrounds

When discussing cultural differences in pain, it is important to remember that the individual health care provider is also embedded in a cultural framework. As Kugelmann et al. [53] explored for chronic pain, the way it is framed in media shapes both how our culture responds to chronic pain and how individuals respond to their own chronic pain – and ultimately how we respond to other cultures [54]. Even within our own culture, we claim to report our pain as accurately as possible, while at the same time believing that others over-report their pain – what has been called the “fundamental pain bias” [54]. For cultures other than our own, the patient’s ethnic background was shown to be associated with the physician’s perception of whether a patient was exaggerating symptoms. The physician's perception of whether a patient is exaggerating was negatively associated with subsequent pain relief [56]. Anderson [57•] even simulated a clinical interaction between a “patient” and “physician” and examined the effect of either (self-identified) racial/ethnic concordance or discordance on the interaction. In non-Hispanic Black/African American patients concordance reduced self-reported and physiological indicators of pain but not in non-Hispanic White patients. In contrast, concordance was associated with increased pain report in those patients. For White patients, no association in either direction could be observed. Importantly, concordance had the greatest impact on pain among patients who reported past experience with or current concern about racial/ethnic discrimination [57•]. Overall, patients often seem to prefer providers who share the same race and/or ethnicity as them, which may lead to improved communication, higher patient satisfaction, and better health outcomes [58]. Racial/ethnic concordance may also help to reduce stigma, such as the assumption among health care providers that minorities are more likely to abuse drugs, which impede unbiased treatment [15]. For example, in a cross-sectional study of Human Immunodeficiency Virus infected patients, Non-White patients were shown to be trusted less by their primary physician despite similar rates of illicit drug use or opioid misuse in comparison to White patients [59]. However, the lack of underrepresented minority (URM) providers makes it difficult for minority group patients to seek care from racially/ethnically concordant clinicians [58]. US studies have also shown that physicians spend less time with Hispanic and Black patients than with Whites [60-62]. These differences persisted even after adjustment for demographics, insurance type, geography, visit intensity and health status [60]. A similar effect was found in women who had undergone cesarean delivery where White women were asked more often about their pain [32]. Ultimately, this led to a inequality in pain relief as these White women were offered more pain medication than the other ethnic groups [32]. In another study, Hispanic and non-Hispanic Black women also experienced disparities in postpartum pain management that could not be explained by less perceived pain [63]. In an attempt to address these pressing issues, increasing racial/ethnic diversity among health care providers could dramatically improve the care of minority group patients [64].

The fact that members of different ethnic groups other than Whites are much more likely to receive only inadequate analgesic treatment has been extensively studied in the US and published in several studies [60•, 65]. A number of papers [65] reported significant differences in pain medication prescription between people from different cultural groups. Overall Black, Hispanic and Asian patients received less pain medication than Whites. Emphasizing the magnitude of the problem, no study was able to conclude that White patients were significantly less likely to receive pain medication [65]. On the other hand, Rosenbloom et al. [66] found no statistically significant race/ethnicity interaction for the administration of opioid analgesia in the emergency room. In general, not only access to analgesics but also to health care in general remains a challenge for minorities, which is beyond of the scope of this review [15].

**Fig. 1:**
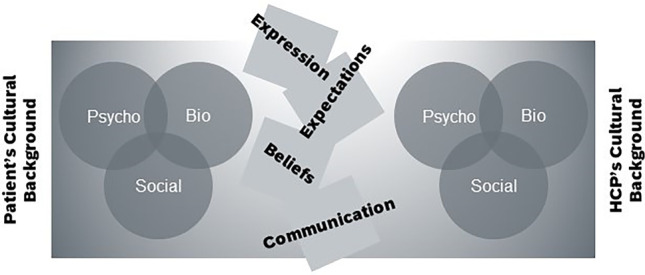
The interrelation of biopsychosocial factors (modified after Fillingim [14]) results in a unique combination of different factors contributing to pain and an individual concept of pain. The individual combination, in turn, shapes the interaction between the patient and the health care provider (HCP). The cultural background of both the patient and the HCP influences all aspects within the biopsychosocial model as well as the interaction

In summary, culture has significant impact on interactions between health care providers and patients on both sides (Fig. 1). Health care professionals should be aware of this influence in order to prevent inequities and biases.

## Impact of Culture on Pain Management and Pain Services

Working toward culturally sensitive medicine should be a priority objective of any health care institution or provider in order to deliver optimal and fair care. Cultural, racial and ethnic differences continue to play a major role in the disparate treatment of acute pain [67]. Meints et al. have comprehensively reviewed patient, provider and system factors that influence racial and ethnic disparities in pain [68]. Further, a recent review by Handtke et al. [69] suggested strategies on how culturally competent healthcare could be provided at an organizational level. For a start, health care professionals should recognize that the concept of pain differs among cultures, which might influence the threshold for seeking help or accepting certain treatments and interventions as well as the choice of coping strategies. Cain et al. [70] recommend the ABCDE model to ascertain the level of cultural influence in the setting of palliative care, which can also prove helpful in the setting of pain management. Health care providers should further recognize that their own beliefs, values and understanding of pain as well as their personal cultural environment shape the way they interact with patients. Narayan [15] has published a set of self-assessment questions that can help health care professionals to determine their own cultural norms concerning pain. Awareness should reflect in the implementation of strategies aimed at a sensitive approach to culturally diverse patients in pain [15, 71]. A mainstay of these concepts is appropriate communication. In pain assessment for example, it includes questions asked in the patient’s language, the use of visual analogs consistent with cultural expectations and open questions to explore the dimensions of pain [15]. We should also recognize that showing and talking about pain might not be acceptable in certain cultures. Nevertheless, these patients must receive appropriate analgesia. Emphasizing the importance of assessing patient perceptions preoperatively, one study shows that preoperative pain- and surgery-related beliefs have a significant impact on the prevalence of acute and chronic post-operative pain, even after controlling other preoperative characteristics such as gender, education and surgery time [72]. Genetic and biological pathway variations also influence pain perception and drug sensitivity, although many of these effects may be the result of increased exposure to stress throughout an individual’s life. Therefore, sociodemographic and psychosocial factors of certain cultures and ethnical/racial backgrounds, which might expose them to increased life stress, should also be considered in the management of pain. Most importantly, cultural competence and sensitivity can be learned through education and training. A recent review highlighted that teaching projects in nursing students that combine multiple competencies, teacher training and continuous transversal projects seem to be most effective [73]. In parallel, existing programs could be ameliorated by including greater exploration of cultural beliefs during assessment, improving accessibility of information about the service and engaging more diverse participants and facilitators [74].

## Consequences

Cultural context is an important factor to consider when caring for a patient in pain. Although only loosely defined, culture integrates a predisposing set of diverse biological, psychological, and social characteristics shared within a group. Individual cultural and ethnic background have been shown to strongly influence the perception, manifestation, and management of pain. Nevertheless, a simple cultural construct can never capture the complexity and true beliefs of an individual patient and carries the risk of stigmatization. Cultural, racial, and ethnic differences continue to play a major role in the disparate treatment of acute pain. Overall, a holistic and culturally sensitive approach is likely to improve pain management outcomes, will better meet the needs of a diverse patient population and help reduce stigma, inequalities and disparities. This requires ongoing education of health care providers and a general awareness that both their own and the patient’s culture significantly influence the relationship.

## Data Availability

Not applicable.

## References

[1] International Association for the Study of Pain. Definition of pain [Internet]. Washington, D.C.: IASP; 2020 [cited 2023 Feb 17]. Available from : https://www.iasp-pain.org/publications/iasp-news/iasp-announces-revised-definition-of-pain/.

[2] Miller ET, Abu-Alhaija DM (2019). Cultural Influences on Pain Perception and Management. Pain Manag Nurs.

[3] American Society of Anesthesiologists Task Force on Acute Pain Management (2012). Practice guidelines for acute pain management in the perioperative setting: an updated report by the American Society of Anesthesiologists Task Force on Acute Pain Management. Anesthesiology.

[4] American Society of Anesthesiologists Task Force on Chronic Pain Management, American Society of Regional Anesthesia and Pain Medicine (2010). Practice guidelines for chronic pain management: an updated report by the American Society of Anesthesiologists Task Force on Chronic Pain Management and the American Society of Regional Anesthesia and Pain Medicine. Anesthesiology.

[5] Smith TJ, Hillner BE (2019). The Cost of Pain. JAMA Netw Open.

[6] Phillips CJ (2009). The Cost and Burden of Chronic Pain. Reviews in Pain.

[7] Fenton BW, Shih E, Zolton J (2015). The neurobiology of pain perception in normal and persistent pain. Pain Management.

[8] Jacob L, Kostev K (2018). Prevalence of pain medication prescriptions in France, Germany, and the UK - a cross-sectional study including 4,270,142 patients. Postgrad Med.

[9] Goesling J, Lin LA, Clauw DJ (2018). Psychiatry and Pain Management: at the Intersection of Chronic Pain and Mental Health. Curr Psychiatry Rep.

[10] Nicholas E. Hagemeier P (2018) Introduction to the Opioid Epidemic: The Economic Burden on the Healthcare System and Impact on Quality of Life. 24:29851449

[11] Chapman WP, Jones CM (1944). Variations in cutaneous and visceral pain sensitivity in normal subjects 1. J Clin Invest.

[12] Miller PK, Van Der Zee S, Elliott D (2022). Pain, Culture and Pedagogy: A Preliminary Investigation of Attitudes Towards “Reasonable” Pain Tolerance in the Grassroots Reproduction of a Culture of Risk. Psychol Rep.

[13] Krahé C, Springer A, Weinman JA, Fotopoulou A (2013). The Social Modulation of Pain: Others as Predictive Signals of Salience – a Systematic Review. Front Hum Neurosci.

[14] Fillingim RB (2017). Individual differences in pain: understanding the mosaic that makes pain personal. Pain.

[15] Narayan MC (2010). Culture’s Effects on Pain Assessment and Management. AJN, American Journal of Nursing.

[16] Lebano A, Hamed S, Bradby H (2020). Migrants’ and refugees’ health status and healthcare in Europe: a scoping literature review. BMC Public Health.

[17] UNESCO. The 2009 UNESCO framework for cultural statistics (FCS) [Internet]. Montreal: UNESCO; 2009 [cited 2023 Feb 17]. Available from: https://unstats.un.org/unsd/statcom/doc10/Bg-FCS-e.pdf.

[18] Palermo TM, Davis KD, Bouhassira D, Hurley RW, Katz JD, Keefe FJ, Schatman M, Turk DC, Yarnitsky D (2023). Promoting Inclusion, Diversity, and Equity in Pain Science. J Pain.

[19] Hoffman KM, Trawalter S, Axt JR, Oliver MN (2016). Racial bias in pain assessment and treatment recommendations, and false beliefs about biological differences between blacks and whites. Proc Natl Acad Sci USA.

[20] American Psychological Association. Racial and ethnic identity [Internet]. Washington, D.C.: APA; 2019 [cited 2023 Mar 13] Available from: https://apastyle.apa.org/style-grammar-guidelines/bias-free-language/racial-ethnic-minorities.

[21] Rahim-Williams B, Riley JL, Williams AKK, Fillingim RB (2012). A Quantitative Review of Ethnic Group Differences in Experimental Pain Response: Do Biology, Psychology and Culture Matter?. Pain Med.

[22] •• Perry M, Baumbauer K, Young EE, Dorsey SG, Taylor JY, Starkweather AR. The Influence of Race, Ethnicity and Genetic Variants on Postoperative Pain Intensity: An Integrative Literature Review. Pain Manag Nurs. 2019;20:198–206. **Comprehensive Review on the influence of race, ethnicity and genetic variants on postoperative pain intensity**. 10.1016/j.pmn.2018.11.002 PMC7841600 31080143

[23] Fillingim RB, Kaplan L, Staud R, Ness TJ, Glover TL, Campbell CM, Mogil JS, Wallace MR (2005). The A118G single nucleotide polymorphism of the μ-opioid receptor gene (OPRM1) is associated with pressure pain sensitivity in humans. J Pain.

[24] Mogil JS (2012). Sex differences in pain and pain inhibition: multiple explanations of a controversial phenomenon. Nat Rev Neurosci.

[25] Valencia C, Smiley A, Giron M, Stacy J, Rodriguez I, Umucu E (2021). Differences in Psychosocial Factors and Experimental Pain Sensitivity Between Hispanics and Non-Hispanic Whites from the U.S.-Mexico Border. Pain Med.

[26] Al-Harthy M, Ohrbach R, Michelotti A, List T (2016). The effect of culture on pain sensitivity. J Oral Rehabil.

[27] Aufiero M, Stankewicz H, Quazi S, Jacoby J, Stoltzfus J (2017). Pain Perception in Latino vs. Caucasian and Male vs. Female Patients: Is There Really a Difference?. WestJEM.

[28] Vina ER, Ran D, Ashbeck EL, Kwoh CK (2018). Natural history of pain and disability among African-Americans and Whites with or at risk for knee osteoarthritis: A longitudinal study. Osteoarthritis Cartilage.

[29] Riddle DL, Slover J, Keefe FJ, Ang DC, Dumenci L, Perera RA (2021). Racial Differences in Pain and Function Following Knee Arthroplasty: A Secondary Analysis From a Multicenter Randomized Clinical Trial. Arthritis Care Res.

[30] Flowers PPE, Schwartz TA, Arbeeva L (2020). Racial Differences in Performance-Based Function and Potential Explanatory Factors Among Individuals With Knee Osteoarthritis. Arthritis Care Res.

[31] Vaughn IA, Terry EL, Bartley EJ, Schaefer N, Fillingim RB (2019). Racial-Ethnic Differences in Osteoarthritis Pain and Disability: A Meta-Analysis. J Pain.

[32] Johnson JD, Asiodu IV, McKenzie CP, Tucker C, Tully KP, Bryant K, Verbiest S, Stuebe AM (2019). Racial and Ethnic Inequities in Postpartum Pain Evaluation and Management. Obstet Gynecol.

[33] Kim HJ, Yang GS, Greenspan JD, Downton KD, Griffith KA, Renn CL, Johantgen M, Dorsey SG (2017). Racial and ethnic differences in experimental pain sensitivity: systematic review and meta-analysis. Pain.

[34] •• Tanner JJ, Johnson AJ, Terry EL, et al. Resilience, pain, and the brain: Relationships differ by sociodemographics. J Neurosci Res. 2021;99:1207–35. **Exporing the link between resilience, pain, brain structural changes and sociodemographic factors**. 10.1002/jnr.24790 PMC8634062 33606287

[35] Dawson LP, Andrew E, Nehme Z (2022). Association of Socioeconomic Status With Outcomes and Care Quality in Patients Presenting With Undifferentiated Chest Pain in the Setting of Universal Health Care Coverage. JAHA.

[36] •• Losin EAR, Woo C-W, Medina NA, Andrews-Hanna JR, Eisenbarth H, Wager TD. Neural and sociocultural mediators of ethnic differences in pain. Nat Hum Behav. 2020;4:517–30. **Association of sociocultural mediators with ethnic differences in pain rating and prevalence**. 10.1038/s41562-020-0819-8 PMC7494052 32015488

[37] Terry EL, Tanner JJ, Cardoso JS (2021). Associations of pain catastrophizing with pain-related brain structure in individuals with or at risk for knee osteoarthritis: Sociodemographic considerations. Brain Imaging Behav.

[38] Bartley EJ, Hossain NI, Gravlee CC (2019). Race/Ethnicity Moderates the Association Between Psychosocial Resilience and Movement-Evoked Pain in Knee Osteoarthritis. ACR Open Rheumatology.

[39] Jimenez N, Galinkin JL (2015). Personalizing Pediatric Pain Medicine: Using Population-Specific Pharmacogenetics, Genomics, and Other –Omics Approaches to Predict Response. Anesth Analg.

[40] Navarro-Prado S, Sánchez-Ojeda M, Marmolejo-Martín J, Kapravelou G, Fernández-Gómez E, Martín-Salvador A (2022). Cultural influence on the expression of labour-associated pain. BMC Pregnancy Childbirth.

[41] • Najem C, Mukhtar NB, Ayoubi F, Meeus M (2021) Religious Beliefs and Attitudes in Relation to Pain, Pain-Related Beliefs, Function, and Coping in Chronic Musculoskeletal Pain: A Systematic Review. Pain Physician. ** Systematic review on pain and religious belief**. 34793635

[42] Razum O, Reiss K, Breckenkamp J, Kaufner L, Brenne S, Bozorgmehr K, Borde T, David M (2017). Comparing provision and appropriateness of health care between immigrants and non-immigrants in Germany using the example of neuraxial anaesthesia during labour: cross-sectional study. BMJ Open.

[43] Mustajoki M, Forsén T, Kauppila T (2018). Pain assessment in native and non-native language: difficulties in reporting the affective dimensions of pain. Scand J Pain.

[44] Holt S, Waterfield J (2018). Cultural aspects of pain: A study of Indian Asian women in the UK. Musculoskeletal Care.

[45] Power S, Bogossian FE, Sussex R, Strong J (2017). Examining the nexus of labour pain and culture using an applied social science framework. Horiz Enferm.

[46] • Lor M, Koleck TA. Patient Race, Ethnicity, Language, and Pain Severity in Primary Care: A Retrospective Electronic Health Record Study. Pain Manag Nurs. 2022;23:385–90. **Study on the impact of language barriers on pain**. 10.1016/j.pmn.2022.01.007 PMC9308623 35260338

[47] Crombez P, Bron D, Michiels S (2019). Multicultural approaches of cancer pain. Curr Opin Oncol.

[48] Chen L-M, Miaskowski C, Dodd M, Pantilat S (2008). Concepts Within the Chinese Culture That Influence the Cancer Pain Experience. Cancer Nurs.

[49] Boring BL, Walsh KT, Nanavaty N, Ng BW, Mathur VA (2021). How and Why Patient Concerns Influence Pain Reporting: A Qualitative Analysis of Personal Accounts and Perceptions of Others’ Use of Numerical Pain Scales. Front Psychol.

[50] Meeus M (2018). Are Pain Beliefs, Cognitions, and BehaviorsInfluenced by Race, Ethnicity, and Culture inPatients with Chronic Musculoskeletal Pain: ASystematic Review. Pain Phys.

[51] Krupić F, Čustović S, Jašarević M, Šadić S, Fazlić M, Grbic K, Samuelsson K. Ethnic differences in the perception of pain: a systematic review of qualitative and quantitative research. Medicinski Glasnik 2019. 10.17392/966-19 10.17392/966-19 30256059

[52] Thong ISK, Tan G, Lee TYC, Jensen MP. A Comparison of Pain Beliefs and Coping Strategies and Their Association with Chronic Pain Adjustment Between Singapore and United States. Pain Med pnw237 2019. 10.1093/pm/pnw237 27694147

[53] Kugelmann R, Watson K, Frisby G (2019). Social representations of chronic pain in newspapers, online media, and film. Pain.

[54] Sullivan MD (2019). Clarifying our cultural contest about chronic pain. Pain.

[55] Boring BL, Ng BW, Nanavaty N, Mathur VA (2022). Over-Rating Pain is Overrated: A Fundamental Self-Other Bias in Pain Reporting Behavior. J Pain.

[56] Miner J (2006). Patient and Physician Perceptions as Risk Factors for Oligoanalgesia: A Prospective Observational Study of the Relief of Pain in the Emergency Department. Acad Emerg Med.

[57] • Anderson SR, Gianola M, Perry JM, Losin EAR. Clinician-Patient Racial/Ethnic Concordance Influences Racial/Ethnic Minority Pain: Evidence from Simulated Clinical Interactions. Pain Med. 2020;21:3109–25. **Investigation of the impact of racial/ethnic concordance (clinician/patient) on pain and communication**. 10.1093/pm/pnaa258 PMC8453614 32830855

[58] Moore C, Coates E, Watson A, de Heer R, McLeod A, Prudhomme A. It’s Important to Work with People that Look Like Me. Black Patients’ Preferences for Patient-Provider Race Concordance. J Racial Ethn Health Disparities 2022;1–13. 10.1007/s40615-022-01435-y PMC9640880 36344747

[59] Moskowitz D, Thom DH, Guzman D, Penko J, Miaskowski C, Kushel M (2011). Is Primary Care Providers’ Trust in Socially Marginalized Patients Affected by Race?. J GEN INTERN MED.

[60] • Ly DP. Racial and Ethnic Disparities in the Evaluation and Management of Pain in the Outpatient Setting, 2006–2015. Pain Med. 2019;20:223–32. **Example of racial and ethnic disparities in both evaluation and management of pain**. 10.1093/pm/pny074 PMC6374136 29688509

[61] Hirsh A, Hollingshead N, Ashburn-Nardo L, Kroenke K (2015). (482) Evidence of racial disparities in time spent with patients in pain. J Pain.

[62] Shen MJ, Peterson EB, Costas-Muñiz R, Hernandez MH, Jewell ST, Matsoukas K, Bylund CL (2018). The Effects of Race and Racial Concordance on Patient-Physician Communication: A Systematic Review of the Literature. J Racial Ethn Health Disparities.

[63] Badreldin N, Grobman WA, Yee LM (2019). Racial Disparities in Postpartum Pain Management. Obstet Gynecol.

[64] Kirksey L, Sorour AA, Duson S, Osman MF, Downing LJ, Ayman A, Rowe V (2023). Racial Diversity and Black Vascular Surgeons in Vascular Surgery Workforce. J Vasc Surg.

[65] Clarke G, Chapman E, Crooks J, Koffman J, Ahmed S, Bennett MI (2022). Does ethnicity affect pain management for people with advanced disease? A mixed methods cross-national systematic review of ‘very high’ Human Development Index English-speaking countries. BMC Palliat Care.

[66] Rosenbloom JM, Burns SM, Kim E, August DA, Ortiz VE, Houle TT (2019). Race/Ethnicity and Sex and Opioid Administration in the Emergency Room. Anesth Analg.

[67] Lee P, Le Saux M, Siegel R, Goyal M, Chen C, Ma Y, Meltzer AC (2019). Racial and ethnic disparities in the management of acute pain in US emergency departments: Meta-analysis and systematic review. Am J Emerg Med.

[68] Meints SM, Cortes A, Morais CA, Edwards RR. Racial and ethnic differences in the experience and treatment of noncancer pain. Pain Manag 9:317–334. 10.2217/pmt-2018-0030 PMC6587104 31140916

[69] Handtke O, Schilgen B, Mösko M (2019). Culturally competent healthcare – A scoping review of strategies implemented in healthcare organizations and a model of culturally competent healthcare provision. PLoS ONE.

[70] Cain CL, Surbone A, Elk R, Kagawa-Singer M (2018). Culture and Palliative Care: Preferences, Communication, Meaning, and Mutual Decision Making. J Pain Symptom Manage.

[71] Davidhizar R, Giger JN (2004). A review of the literature on care of clients in pain who are culturally diverse. Int Nurs Rev.

[72] Wang Y, Liu Z, Chen S, Ye X, Xie W, Hu C, Iezzi T, Jackson T (2018). Pre-surgery beliefs about pain and surgery as predictors of acute and chronic post-surgical pain: A prospective cohort study. Int J Surg.

[73] Gradellini C, Gómez-Cantarino S, Dominguez-Isabel P, Molina-Gallego B, Mecugni D, Ugarte-Gurrutxaga MI (2021). Cultural Competence and Cultural Sensitivity Education in University Nursing Courses. A Scoping Review Front Psychol.

[74] Bull E, Young D, Etchebarne A, Malpus Z (2023). Understanding ethnic minority service user experiences of being invited to and attending group pain programmes: A qualitative service evaluation. Br J Pain.

